# Effects of seaweed extracts on in vitro rumen fermentation characteristics, methane production, and microbial abundance

**DOI:** 10.1038/s41598-021-03356-y

**Published:** 2021-12-16

**Authors:** Youyoung Choi, Shin Ja Lee, Hyun Sang Kim, Jun Sik Eom, Seong Uk Jo, Le Luo Guan, Jakyeom Seo, Hanbeen Kim, Sang Suk Lee, Sung Sill Lee

**Affiliations:** 1 Division of Applied Life Science (BK21), Gyeongsang National University, Jinju, 52828 Republic of Korea; 2 Institute of Agriculture and Life Science (IALS), Gyeongsang National University, Jinju, 52828 Republic of Korea; 3 Institute of Agriculture and Life Science and University-Centered Labs, Gyeongsang National University, Jinju, 52828 Republic of Korea; 4 Department of Agricultural, Food and Nutritional Science, University of Alberta, Edmonton, AB T6G 2P5 Canada; 5 Department of Animal Science, Life and Industry Convergence Research Institute, Pusan National University, Miryang, 50463 Republic of Korea; 6 Ruminant Nutrition and Anaerobe Laboratory, Department of Animal Science and Technology, Sunchon National University, Sunchon, Republic of Korea

**Keywords:** Plant sciences, Zoology, Environmental sciences

## Abstract

Several seaweed extracts have been reported to have potential antimethanogenic effects in ruminants. In this study, the effect of three brown seaweed species (*Undaria pinnatifida*, *UPIN*; *Sargassum fusiforme*, *SFUS*; and *Sargassum fulvellum*, *SFUL*) on rumen fermentation characteristics, total gas, methane (CH_4_), carbon dioxide (CO_2_) production, and microbial populations were investigated using an in vitro batch culture system. Seaweed extract and its metabolites, total flavonoid and polyphenol contents were identified and compared. For the in vitro batch, 0.25 mg∙mL^−1^ of each seaweed extract were used in 6, 12, 24, 36 and 48 h of incubation. Seaweed extract supplementation decreased CH_4_ yield and its proportion to total gas production after 12, 24, and 48 h of incubation, while total gas production were not significantly different. Total volatile fatty acid and molar proportion of propionate increased with *SFUS* and *SFUL* supplementation after 24 h of incubation, whereas *UPIN* was not affected. Additionally, *SFUS* increased the absolute abundance of total bacteria, ciliate protozoa, fungi, methanogenic archaea, and *Fibrobacter succinogenes*. The relative proportions of *Butyrivibrio fibrisolvens*, *Butyrivibrio proteoclasticus,* and *Prevotella ruminicola* were lower with seaweed extract supplementation, whereas *Anaerovibrio lipolytica* increased. Thus, seaweed extracts can decrease CH_4_ production, and alter the abundance of rumen microbial populations.

## Introduction

Methane (CH_4_) is the second largest contributor to greenhouse gas (GHG) emissions after carbon dioxide (CO_2_) and has a global warming potential (GWP) approximately 28 times greater than that of CO_2_^1,2^. Ruminants produce CH_4_ as a metabolic end-product of enteric fermentation in the rumen, representing between 2 and 12% of the gross energy intake^3^. Research on strategies to reduce CH_4_ production is necessary owing to the global awareness and threat.

Although numeral feed additives have been used to decrease CH_4_ production by manipulating the ruminal microbial fermentation, seaweed has shown to be some of the most promising worldwide^4–7^. Seaweed has rich and diverse bioactive compounds, particularly halogenated and polyphenolic metabolites that can suppress methanogenesis in the rumen^8–11^. A species of red seaweed, *Asparagopsis taxiformis* (*A. taxiformis*), including bromoform and dibromochloromethane, specifically inhibits enzymatic activities by binding to vitamin B_12_^12^; this is chemically similar to the coenzyme F430, a cofactor needed for methanogenesis^13^. A species of brown seaweed is the only species to accumulate a variety of polyphenol compounds (e.g., phlorotannins) as an adaptive defense strategy against stress conditions and herbivory^14,15^. A previous study reported that phlorotannins purified from *Ascophyllum nodosum* (*A. nodosum*), a species of brown seaweed, had an antimethanogenic effect without affecting ruminal microbial fermentation^11^. However, the exact mechanism has not yet been understood thus far. Notably, phlorotannins can induce an antimicrobial effect by inactivating extracellular enzymes and proteins necessary for the growth and metabolism of microorganisms^16^. Due to the similarity of chemical structures between phlorotannins and terrestrial tannin, the antimicrobial effect of phlorotannins against rumen methanogens may be worth further exploration^11^.

The current study consider three brown seaweed species (*Undaria pinnatifida*, *UPIN*; *Sargassum fusiforme*, *SFUS*; and *Sargassum fulvellum*, *SFUL*) that were selected based on cultivation potential, biochemical profile, and sustainability as animal feed in ruminant production^17–19^. In addition, our previous in vitro batch culture studies classified dried *UPIN*, *SFUS*, and *SFUL* as consisting of minerals, heavy metals, and metabolites, and also found that the three seaweeds enhanced rumen volatile fatty acid (VFA) concentrations when fed at 1% dry matter (DM) content^17–19^. Moreover, Li et al.^14^ reported that *UPIN*, *SFUS*, and *SFUL*, accumulate phlorotannins with health beneficial biological activities, which in some cases can demonstrate antimicrobial and antimethanogenic effects in ruminants. A reduction in CH_4_ production was observed at the rate of 1% DM of *SFUS*, but not for *UPIN* and *SFUL*. Seaweed in the form of an extract supplemented at 0.25 mg∙mL^−1^ with only timothy hay, exhibited a greater reduction in CH_4_ production after 48 h of incubation^20^. A recent study reported that the supplementation of the red seaweed *A. taxiformis* reduced the rumen methanogens abundance along with a CH_4_ reduction via an in vitro continuous culture system^9^. In contrast, brown seaweed (*A. nodosum* and *Laminaria digitate* (*L. digitata*)) supplementation did not affect the abundance of rumen methanogens, fungi, and total bacteria^15^. However, the impact of *UPIN*, *SFUS*, and *SFUL* on the rumen microbial abundance is still not fully understood.

Therefore, we hypothesize that the supplementation of three different seaweed extracts would reduce enteric CH_4_ production and enhance rumen fermentation characteristics. The main objectives of this study were to: (1) identify the metabolites and potential antioxidants (including the flavonoid and phenol content) of the three brown seaweed extracts; (2) determine if the polyphenolic compounds in the seaweed extracts reduced enteric CH_4_ and CO_2_ production; (3) examine the effect of seaweed extracts on the abundance of rumen bacterial, ciliate protozoal, fungal and methanogenic archaea using real-time polymerase chain reaction (qPCR); and (4) investigate the rumen fermentation characteristics and mode of action of seaweed extracts using in vitro batch culture.

## Results

### Metabolite, total flavonoid and polyphenol profiles of seaweed extracts

**Figure 1:**
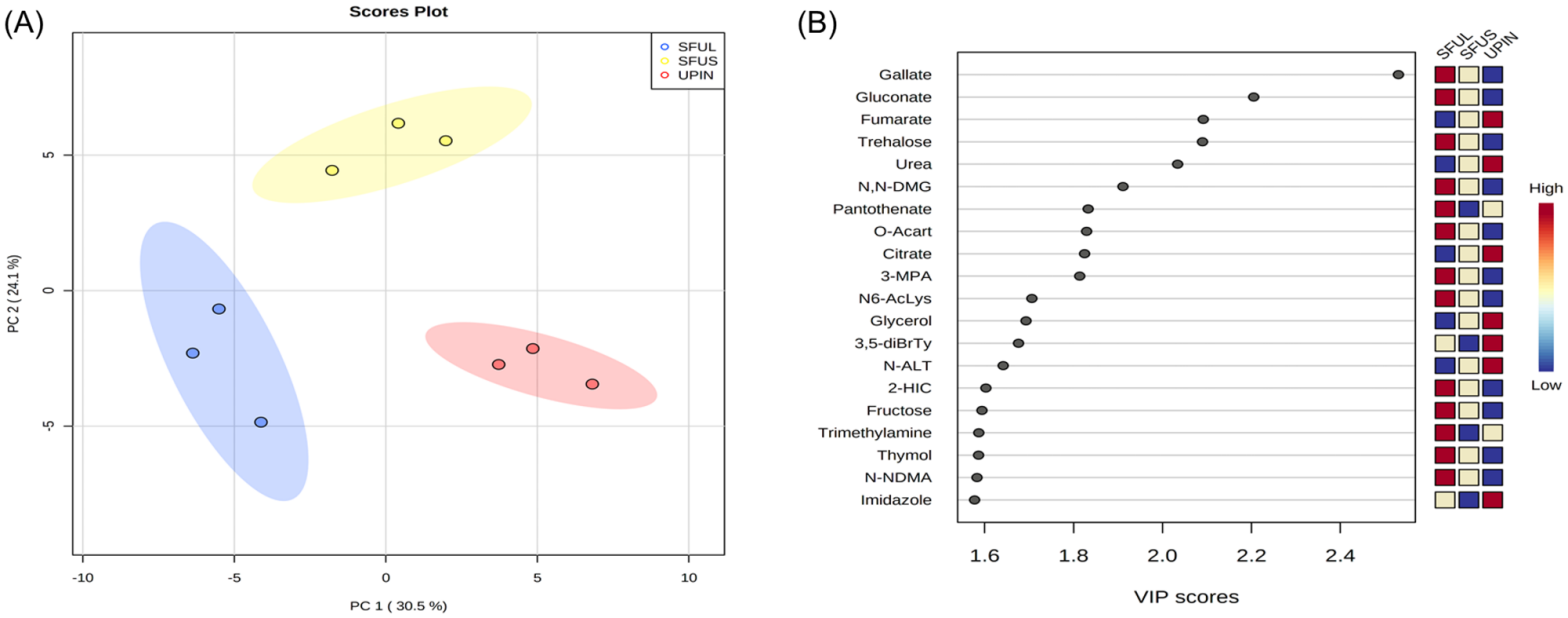
Multivariate score plots for brown seaweed extracts. (**A**) Principal component analysis (PCA) score plot, ellipses represent 95% confidence intervals. (**B**) Variable importance in projection (VIP) scores of the top 20 metabolites with VIP scores > 1.5. *UPIN* (red ellipse), *Undaria pinnatifida*; *SFUS* (yellow ellipse), *Sargassum fusiforme*; *SFUL* (blue ellipse), *Sargassum fulvellum*; 2-HIC, 2-hydroxyisocaproate; 3,5-diBrTy, 3,5-dibromotyrosine; 3-MPA, 3-methyladipate; N6-AcLys, N6-acetyllysine; N-ALT, N-acetyltyrosine; N-NDMA, N-nitrosodimethylamine; N,N-DMG, N,N-dimethylglycine; O-Acart, O-acetylcarnitine.

^1^H-NMR analysis of three seaweed extracts (*UPIN*, *SFUS*, and *SFUL*) identified 149 metabolites in the three seaweed extracts (Fig. 1 and Table 1). However, only 24 metabolites (including guanidoacetate, ethylene glycol, alanine, galactose, and *inter alia*) were classified into four chemical classes (organic acids, carbohydrates, amino acids, and lipids). The PCA plot showed separated clusters among the three seaweed extracts and revealed differences that were considerably separated in PC 1 (30.5%), and PC 2 (24.1%) in PCA (Fig. 1A). Further PLS-DA analysis of relative intensities of the metabolites revealed the significant differences of identified metabolites in seaweed extracts with 20 of them being significantly different (VIP score > 1.5) (Fig. 1B). We found gallate in the extract that obtained highest VIP score, which possesses antioxidant properties, and 14 phenolic metabolites were detected in *SFUL* (69.20 ± 7.86 µM) and *SFUS* (2.10 ± 0.06 µM) but not in *UPIN*. The total flavonoid and polyphenol contents are shown in Table 1. Total flavonoid content varied between species and was highest for *SFUL* (25.21 ± 1.72 mg CE/g) followed by *UPIN* (1.66 ± 0.26 mg CE/g) and lowest in *SFUS* (0.49 ± 0.12 mg CE/g). The total polyphenol content was highest in *SFUL* (5.77 ± 0.07 mg GAE/g) followed by *SFUS* (1.89 ± 0.05 mg GAE/g) and lowest in *UPIN* (1.59 ± 0.16 mg GAE/g). In the present study, *SFUL* had higher total flavonoid and polyphenol contents than the other seaweed extracts.

**Table 1:** Metabolite concentrations and total flavonoid and polyphenol contents of brown seaweed extracts.

Metabolites^a^ (µM)	Class^b^	*UPIN*	*SFUS*	*SFUL*
**Analyzed by **^**1**^**H-NMR**				
Guanidoacetate (mM)	OA	6.59 ± 0.32	6.56 ± 0.19	6.29 ± 0.24
Ethylene glycol (mM)	Lipids	2.30 ± 0.11	2.32 ± 0.15	2.11 ± 0.10
Alanine (mM)	AA	0.34 ± 0.02	0.77 ± 0.01	0.54 ± 0.01
Galactose	CHO	87.00 ± 23.27	186.63 ± 55.69	199.90 ± 5.96
Mannose	CHO	106.76 ± 22.32	72.20 ± 35.31	198.25 ± 18.57
Fucose	CHO	77.86 ± 37.41	122.70 ± 38.16	154.55 ± 1.10
N-NDMA	OA	37.10 ± 1.55	142.30 ± 40.38	217.70 ± 2.48
Formate	OA	245.36 ± 7.32	69.86 ± 1.71	118.90 ± 5.13
Acetate	OA	162.36 ± 7.45	18.76 ± 2.62	111.96 ± 3.67
Malonate	OA	27.30 ± 11.80	42.45 ± 11.88	101.30 ± 29.65
Lactulose	CHO	27.00 ± 7.42	73.60 ± 7.97	99.76 ± 6.11
**Phenolic metabolite**				
2-Hydroxyphenylacetate	BZA	–	2.45 ± 0.12	3.87 ± 0.94
2-Phenylpropionate	BZA	4.65 ± 1.02	–	11.20 ± 0.16
3-Hydroxyphenylacetate	BZA	10.13 ± 2.64	1.87 ± 0.29	15.20 ± 0.16
3-Phenylpropionate	BZA	5.80 ± 0.86	16.13 ± 1.53	–
4-Hydroxyphenylacetate	BZA	–	25.50 ± 2.60	22.13 ± 5.43
4-Hydroxyphenyllactate	BZA	5.95 ± 0.28	–	79.30 ± 7.02
Gallate	BZA	–	2.10 ± 0.06	69.20 ± 7.86
Syringate	BZA	3.00 ± 0.57	0.70 ± 0.02	6.73 ± 0.88
Tartrate	BZA	28.70 ± 0.92	9.97 ± 0.24	23.95 ± 2.90
Homovanillate	BZA	1.70 ± 0.61	1.37 ± 0.22	2.63 ± 1.02
Vanillate	BZA	1.70 ± 0.57	1.23 ± 0.09	1.87 ± 0.37
Caffeine	OA	1.15 ± 0.28	1.05 ± 0.04	2.30 ± 0.55
Ferulate	OA	2.00 ± 0.49	0.70 ± 0.01	3.57 ± 0.87
**Analyzed by optical density**				
Total flavonoid (mg CE/g)		1.66 ± 0.26	0.49 ± 0.12	25.21 ± 1.72
Total polyphenol (mg GAE/g)		1.59 ± 0.16	1.89 ± 0.05	5.77 ± 0.07

All values represent the mean ± SEM (n = 3).

SEM Standard error of the mean, *UPIN Undaria pinnatifida*, *SFUS Sargassum fusiforme*, *SFUL Sargassum fulvellum*, CE Catechin equivalent, GAE Gallic acid equivalent.

^a^Metabolite abbreviations: NDMA, N-Nitrosodimethylamine; –, Not detected.

^b^Class abbreviations: OA, organic acids; CHO, carbohydrates; AA, amino acids; BZA, benzoic acids.

### Effects of seaweed extracts on rumen fermentation characteristics

**Figure 2:**
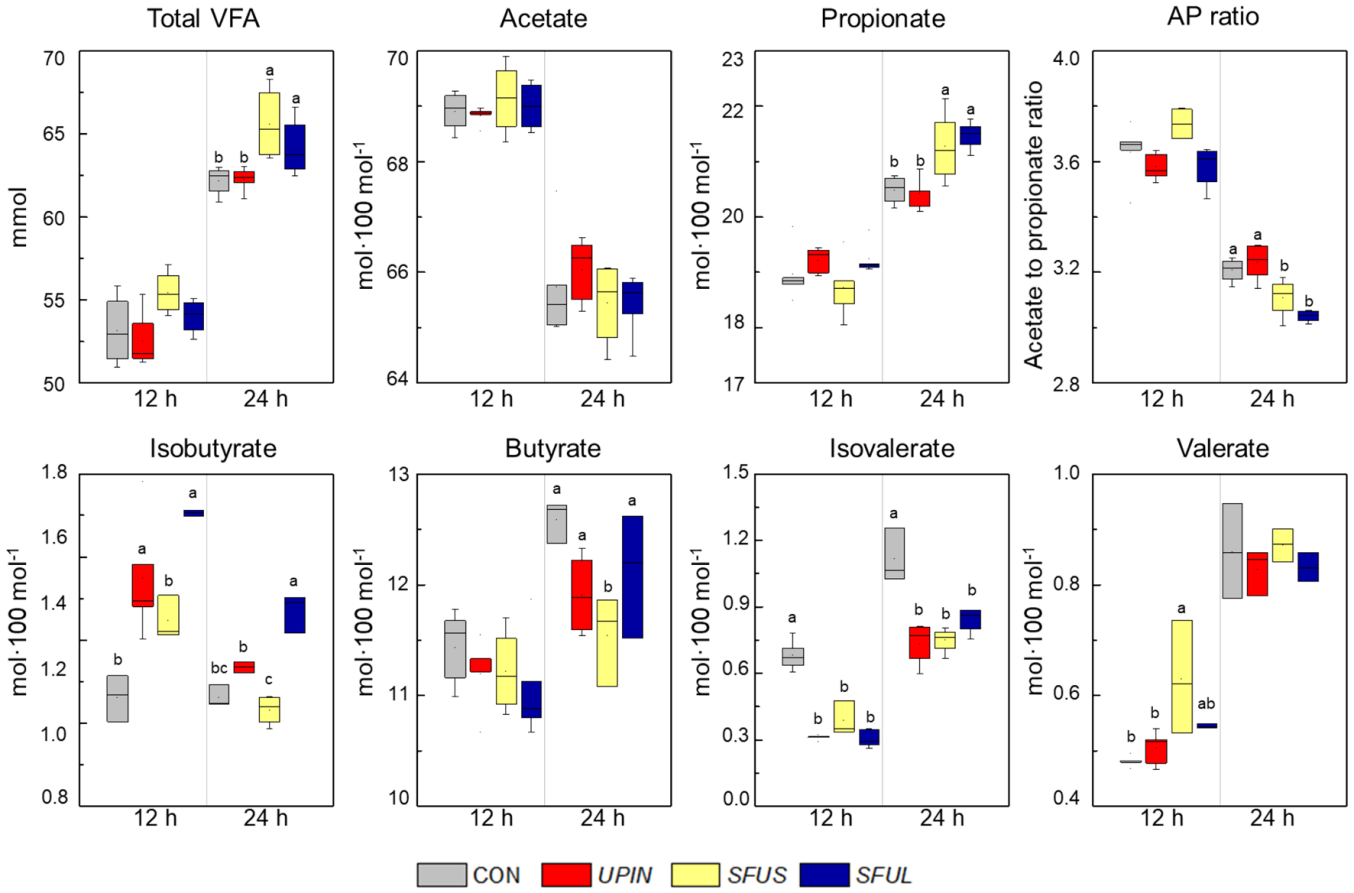
Effects of brown seaweed extracts on volatile fatty acid production after 12 and 24 h of in vitro incubation. CON (gray box), without seaweed extracts; *UPIN* (red box), *Undaria pinnatifida*; *SFUS* (yellow box), *Sargassum fusiforme*; *SFUL* (blue box), *Sargassum fulvellum*; Data were analyzed using seaweed extracts dose amount: 0.25 mg-mL^−1^. Error bars are standard error of the mean. ^a,b^Means (n = 5) with different superscript letters indicate a significant difference (*P* < 0.05).

Rumen fermentation characteristics are shown in Table 2 and Fig. 2. Compared to the CON, *UPIN*, *SFUS*, and *SFUL* supplementation resulted in the pH being greater (*P* = 0.001) at 6 h of early incubation time. The apparent DM digestibility was estimated by a nylon bag, and it tended to be affected by supplementation, wherein *SFUS* was lower (*P* = 0.059) than CON at 12 h of incubation, and no differences were observed afterward. The ammonia nitrogen (NH_3_-N) concentration was considerably lower by supplementation with *UPIN*, *SFUS*, and *SFUL* compared with CON from 12 h up to 48 h of incubation. The most pronounced decrease in NH_3_-N concentration was observed (*P* < 0.001) at 12 h of incubation. The concentration of total VFA did not differ among treatments at 12 h of incubation. However, it was considerably higher (*P* < 0.05) with *SFUS* and *SFUL* than CON at 24 h of incubation. None of the seaweed extracts affected the molar percentage of acetate concentration at both 12 and 24 h of incubation. *SFUS* and *SFUL* showed a significantly higher (*P* < 0.001) molar percentage of propionate at 24 h of incubation. A tendency (*P* = 0.057) indicated that *SFUS* showed a lower molar percentage of butyrate at 24 h of incubation, whereas *UPIN* and *SFUL* did not affect the butyrate molar percentage in the cultures. The molar percentage of isobutyrate increased by with *SFUL* supplementation at both 12 (*P* < 0.01) and 24 h (*P* < 0.001) of incubation compared to the CON. In contrast, the molar percentage of isovalerate was greater in CON among treatments at both 12 (*P* < 0.001) and 24 h (*P* < 0.001) of incubation. *SFUS* resulted in a significantly higher (*P* < 0.01) molar percentage of valerate at 12 h of incubation but did not differ among the treatments at 24 h of incubation. As a result, the acetate to propionate (AP) ratio was lower (*P* = 0.054, *P* < 0.001) for *SFUL* at 12 and 24 h of incubation.

**Table 2:** Effects of brown seaweed extracts on pH, DM digestibility, and NH_3_-N concentration in vitro incubation.

Parameters	CON	Treatments^1^			SEM^2^	*P* value
		*UPIN*	*SFUS*	*SFUL*		
**pH**						
6 h	6.96^b^	7.04^a^	7.03^a^	7.05^a^	0.01	0.001
12 h	6.58	6.60	6.66	6.65	0.03	0.189
24 h	6.26	6.28	6.24	6.31	0.03	0.410
36 h	6.07	6.08	6.08	6.17	0.03	0.134
48 h	5.99	5.97	5.96	5.98	0.02	0.927
**DM digestibility (%)**						
6 h	28.96	27.24	27.12	28.70	1.07	0.506
12 h	39.75	38.65	35.60	40.85	1.25	0.059
24 h	48.50	48.28	50.65	49.00	1.22	0.554
36 h	57.12	56.80	55.20	56.45	0.94	0.527
48 h	58.64	58.36	59.72	59.28	0.92	0.721
**NH**_**3**_**-N concentration (mg∙dL**^**−1**^**)**						
6 h	9.94	10.05	9.62	9.49	0.36	0.634
12 h	11.67^a^	9.54^b^	9.17^b^	8.59^b^	0.28	< 0.001
24 h	16.46^a^	15.18^ab^	13.18^c^	14.36^bc^	0.43	< 0.001
36 h	21.35^a^	18.77^b^	18.98^b^	20.00^ab^	0.41	0.002
48 h	24.77^a^	24.27^a^	21.79^b^	22.25^b^	0.50	< 0.001

CON Without seaweed extracts, *UPIN Undaria pinnatifida*, *SFUS Sargassum fusiforme*, *SFUL Sargassum fulvellum*, DM Dry matter, NH_3_-N Ammonia nitrogen.

^1^Data were analyzed using seaweed extracts dose amount: 0.25 mg-mL^−1^.

^2^SEM, standard error of the mean.

^a,c^Means (n = 5) in a row followed by different superscript letters are significantly different (*P* < 0.05).

### Effects of seaweed extracts on total gas, CH_4_, and CO_2_ production

**Figure 3:**
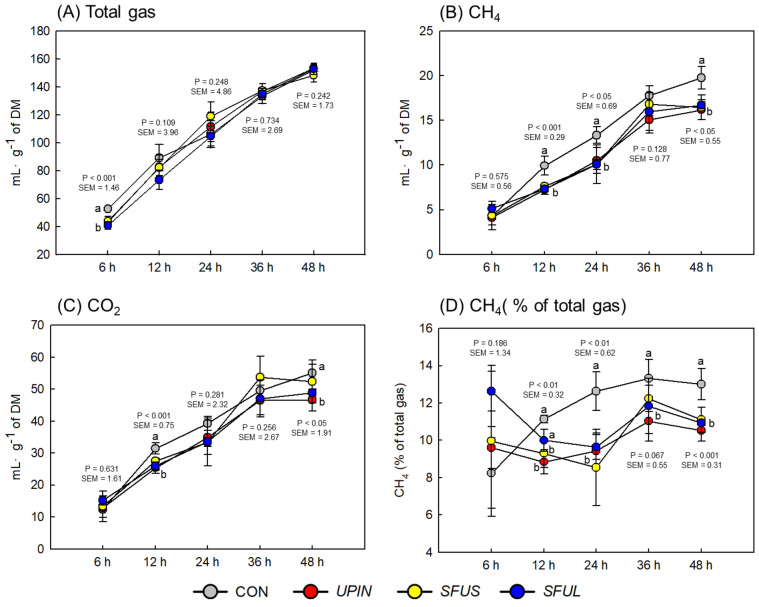
Effects of brown seaweed extracts on (**A**) total gas, (**B**) CH_4_, (**C**) CO_2_ production, and (**D**) total gas (% of CH_4_) in vitro. CON (gray circle), without seaweed extracts; *UPIN* (red circle), *Undaria pinnatifida*; *SFUS* (yellow circle), *Sargassum fusiforme*; *SFUL* (blue circle), *Sargassum fulvellum*; Data were analyzed using seaweed extracts dose amount: 0.25 mg-mL^−1^. Error bars are standard error of the mean. ^a,b^Means (n = 5) with different superscript letters indicate a significant difference (*P* < 0.05).

Total gas production was significantly lower (*P* < 0.001) in *UPIN*, *SFUS*, and *SFUL* supplementation compared with CON at an early incubation time (Fig. 3). The CH_4_ production lowered gradually up to 48 h during the incubation for all treatments. Compared to CON, the most pronounced reduction was observed in the *UPIN*, *SFUS*, and *SFUL* supplementation at 12 (reduction of 26.8%, 23.4%, 26.3%, *P* < 0.001, respectively), and 24 h (reduction of 21.3%, 24.4%, 24.6%, *P* < 0.05, respectively) of incubation. Compared with CON, the CH_4_ proportion to total gas production by all seaweed extract supplementations was significantly lower at 12 (*P* < 0.01), 24 (*P* < 0.01), and 48 h (*P* < 0.001) of incubation. The CO_2_ production tended to be significantly lower with *UPIN*, *SFUS*, and *SFUL* supplementation compared with CON at 12 (*P* < 0.001) and 48 h (*P* < 0.05) of incubation.

### Effects of seaweed extracts on the abundance of microbial community

Regarding the microbial counts, there was no significant change in the absolute value of total bacteria among the treatment and CON (Table 3). Compared to CON, the populations of both ciliate protozoa (*P* < 0.01) and fungi (*P* < 0.01) were significantly higher with *SFUS* supplementation, while *UPIN* and *SFUL* supplementation were significantly lower. None of the seaweed extracts decreased (*P* < 0.05) the abundance of methanogenic archaea, which the microbial population directly responsible for rumen CH_4_ production, rather *SFUS* supplementation resulted in a significantly higher proportion than CON. In the relative populations of fiber-degrading bacterial species both *Fibrobacter succinogenes* and *Ruminococcus flavefaciens* were significantly higher in *SFUS* (*P* < 0.01) and *SFUL* (*P* < 0.05) supplementation, while *Ruminococcus albus* was not affected by any of the treatments. There was a tendency (*P* = 0.092) that *Butyrivibrio fibrisolvens* abundance reduced in *SFUS* and *SFUL* supplementation compared to CON, while the abundance of *Butyrivibrio proteoclasticus* was not affected by any of the treatments. In contrast, all the treatments had a significantly higher (*P* < 0.01) abundance of *Anaerovibrio lipolytica*, which includes lipolytic bacteria. Except for *UPIN* supplementation, both *SFUS* and *SFUL* had a significantly lower (*P* < 0.001) abundance of *Prevotella ruminicola*, which includes proteolytic bacteria.

**Table 3:** Effects of brown seaweed extracts on microbial abundance after 24 h of in vitro incubation.

Parameters	CON	Treatments^1^			SEM^2^	*P* value
		*UPIN*	*SFUS*	*SFUL*		
**Absolute abundance**						
Total bacteria	3.59	2.77	3.95	3.12	0.30	0.159
Ciliate protozoa	6.24^a^	2.87^b^	7.86^a^	4.01^b^	0.54	0.001
Fungi	1.14^b^	1.12^b^	3.62^a^	2.13^b^	0.26	0.007
Methanogenic archaea	0.92^b^	1.12^b^	1.85^a^	1.16^b^	0.11	0.017
**Relative abundance, % of total bacteria**						
*Fibrobacter succinogenes*	8.51^b^	10.59^b^	13.48^a^	10.16^b^	0.79	0.003
*Ruminococcus albus*	0.27	0.31	0.28	0.28	0.01	0.101
*Ruminococcus flavefaciens*	0.67^b^	0.67^b^	0.64^b^	0.77^a^	0.03	0.011
*Butyrivibrio fibrisolvens*	2.36	2.55	2.13	2.03	0.14	0.092
*Butyrivibrio proteoclasticus*	0.04	0.03	0.03	0.03	0.00	0.146
*Prevotella ruminicola*	31.52^a^	25.79^a^	15.07^b^	12.42^b^	2.15	< 0.001
*Anaerovibrio lipolytica*	0.33^b^	1.03^a^	0.66^ab^	0.83^a^	0.10	0.003

CON Without seaweed extracts, *UPIN Undaria pinnatifida*, *SFUS Sargassum fusiforme*, *SFUL Sargassum fulvellum.*

Total bacteria, × 10^10^ copies∙mL^−1^ of rumen fluid; Ciliate protozoa, × 10^9^ copies∙mL^−1^ of rumen fluid; Fungi, × 10^6^ copies∙mL^−1^ of rumen fluid, Methanogenic archaea, × 10^10^ copies∙mL^−1^ of rumen fluid.

^1^Data were analyzed using seaweed extracts dose amount: 0.25 mg-mL^−1^.

^2^SEM, standard error of the mean.

^a,b^Means (n = 3) in a row followed by different superscript letters are significantly different (*P* < 0.05).

## Discussion

Supplementation of seaweed extracts in ruminant diets has been reported to alter digestion, fermentation characteristics, proteolysis, and microbial communities in the rumen^9,18,21–23^. Polyphenols in seaweeds (Table 1) protect proteins from degradation and improve the efficiency of nitrogen use in ruminants by increasing the amount of by-pass protein and reducing rumen fiber degradation by decreasing the attachment of microbes to feed particles^24^. In the present study, supplementation with seaweed extracts did not affect DM digestibility but lowered the NH_3_-N concentration and AP ratios (Table 2, Fig. 2). This could be attributed to the polyphenolic content in seaweed extracts, which can decelerate ruminal proteolysis, peptidolysis, and deamination^7^. The reduced molar proportion of isovalerate supports this premise^7,10^. Isovalerate is produced by microbial deamination and the decarboxylation of leucine in the rumen^25^; the polyphenolic content could possibly reduce this process. This result is also supported by the higher abundance of fibrolytic bacterial populations, including *F. succinogenes* and *R. flavefaciens* (Table 3) because isovalerate is a well-known requirement for their growth^26^. In addition, the lower abundance of proteolytic bacteria, namely *B. fibrisolvens*, *B. proteoclasticus*, and *P. ruminocola* might also support this result.

In the present study, seaweed extract supplementation induced an evident shift in the VFA pattern compared with the CON (Fig. 2). Notably, *SFUS* and *SFUL* supplementation caused a shift in the ruminal fermentation to a greater molar percentage of propionate without affecting the molar percentage of acetate. Wettstein et al.^27^ reported that decreased rumen methanogenesis sometimes shifts rumen fermentation from acetate to propionate because the propionate synthesis pathway is favored rather than acetate synthesis pathway. Generally, CH_4_ formation in the rumen is regarded as a syntrophy between H_2_-producing microbes and H_2_-consuming methanogens^28^; the manipulation of the H_2_ sink plays an important role in mitigating CH_4_ in the rumen. The major metabolic H_2_ sink strategy for CH_4_ mitigation is the enhancement of propionate production because it acts as a sink for H_2_ and reduces the availability of H_2_ for methanogens^29^. Becker et al.^30^ reported that flavan-3-ol, ( +)-catechin has the potential to be an alternative H_2_ sink for CH_4_ precursors without reducing the VFA production. However, contrary results were obtained in the present study, as *SFUS* and *SFUL* had a greater VFA production. Further analysis of catechin from seaweed extracts and an investigation of the relationship between VFA production and CH_4_ formation in the rumen fluid using an in vitro batch culture system is necessary to clarify the mechanism by which seaweed extracts alter rumen fermentation. The present study showed that both *SFUS* and *SFUL* supplementation had the highest molar percentage of propionate, even though it presented a low population of *P. ruminicola* (Table 3), a major propionate-producing bacterial species in the rumen^31^. A possible explanation for the greater molar percentage of propionate is the high concentration of lipid class metabolites (e.g., ethylene glycol) in seaweed extracts and the higher relative abundance of *A. lipolytica*, an important rumen bacterium for lipid hydrolysis^32^. Our data indicate that the notable scores in *SFUS* and *SFUL* were fumarate and glycerol, respectively. Fumarate can be converted into propionate, and glycerol (a major source of lipid metabolism) is rapidly fermented to propionate in the rumen^33^. However, a limitation in this study was the lack of members of the ruminal microbiome that contribute to the propionate synthesis pathway. Physiological and ecological studies employing metagenomics approaches on rumen bacteria are needed to determine if the seaweed extracts directly affect succinate- and propionate- producing bacteria at the genus or species level in the rumen. This may further support our speculation that seaweed extracts have the potential to increase propionate production. Nevertheless, the type of amino acids, carbohydrates, and preformed organic acids measured in seaweed extracts may affect the molar proportion of accumulated VFAs. Taken together, the decreased AP ratio suggests that metabolic H_2_ is, at least in part, redistributed to propionate, which may partly explain the CH_4_ reduction observed in this study.

There was a trend for a slight increase in total gas production with the supplementation of seaweed extracts, possibly owing to the increase in fiber-degrading bacteria in the seaweed extracts (Table 3, Fig. 3). The novel finding in our study is that supplementation with seaweed extracts caused a 5.4% to 26.8% suppression in CH_4_ production up to 48 h incubation, without compromising DM digestibility or total VFA production. Moreover, the proportion of CH_4_ to total gas production was suppressed by seaweed extract supplementation. The CH_4_ is produced in the rumen and hindgut of ruminants by a group of methanogenic archaea, which are estimated to account for 0.3–3.3% of the rumen microbial population^34,35^. Most of them use H_2_ and CO_2_ produced by some fermentative members of the rumen microbes to produce CH_4_ through the hydrogenotrophic pathway^36^. With significantly lower CH_4_ production, CH_4_-producing microbes (ciliate protozoa and methanogenic archaea) were expected to be less with seaweed extract supplementation. However, *SFUS* supplementation resulted in a larger abundance of ciliate protozoa, and methanogenic archaea, and the total bacteria, fungi, fibrolytic bacteria, and *A. lipolytica*, which may explain the high VFA production compared with CON and other seaweed extracts (Table 3, Fig. 2). The supplementation of *UPIN* and *SFUL* significantly reduced the abundance of ciliate protozoa but did not affect methanogenic archaea. However, these findings agree with the in vitro findings reported by Molina-Alcaide et al.^37^, who suggested that different seaweed species may have variable effects on the abundance of ciliate protozoa. Similarly, according to Henderson et al.^36^, ciliate protozoa and methanogenic archaea are not always correlated, even if they have a mutualistic relationship that enhances CH_4_ formation in the rumen. This indicates that there are also different partner specificities within archaeal and protozoal species. Similar and contradictory results were observed in the in vivo experiment performed in this study, where ciliate protozoal abundance increased via brown seaweed species *A. nodosum*, but total bacteria abundance and DM digestibility were reduced. The seaweed species and the experimental system (in vitro vs. in vivo) were different in these studies, thus, suggesting that an in vivo system would enhance future studies in evaluating the antimethanogenic effects of different seaweed extracts on ciliate protozoa^38^.

Another factor that could affect rumen microbial abundance was linked to the properties of total flavonoids, polyphenols and phenolic metabolites (Table 1) of the three seaweed species, which act as antimicrobial. For example, it has been reported that polyphenolic compounds of seaweed (e.g., phlorotannins) can reduce CH_4_ production in ruminants^22^. However, it is unclear whether this directly affects polyphenolic compounds on CH_4_-producing microbes, including ciliate protozoa and methanogenic archaea. A previous in vitro batch culture study^11^ showed that when phlorotannins (derived from *A. nodosum*) was added to rumen fluid, the NH_3_-N concentration was lower than that without the addition of phlorotannins (Table 2). This result concurs with the present study, in which seaweed extract supplementation reduced the NH_3_-N concentration. The abundance of other proteolytic bacteria (e.g., *Prevotella bryantii*) was greater when phlorotannins was added, despite a significant reduction in NH_3_-N concentration. In contrast, the present study found that all three seaweed extracts lowered the abundance of proteolytic bacteria (*B. fibrisolvens*, *B. proteoclasticus*, and *P. ruminocola*) (Table 3). Thus, our results indicate that the polyphenol content from *UPIN*, *SFUS*, and *SFUL* also have a function in the formation of protein-phenol complexes and discrepancies in the proteolytic bacteria abundance may be partly attributable to the different types of polyphenolic compounds produced by the interspecies difference of seaweed. Nonetheless, additional research is needed to elucidate the mechanism related to CH_4_ production and/or rumen microbial abundance to explain the effects of polyphenols from seaweed-species.

Another finding of this study was that the interaction between seaweed and basal substrates plays an important role in the effectiveness of rumen methanogenesis^23^. Indeed, in our previous research^20^, we found that the supplementation of timothy hay with five, brown seaweed extracts (*Ecklonia stolonifera*, *Eisenia bicyclis*, *UPIN*, *SFUS*, and *SFUL*) decreased CH_4_ production only after long-term in vitro incubation (48 h). However, in the present study we found that CH_4_ production was effectively reduced after short-term to long-term in vitro incubation (12, 24, and 48 h) (Fig. 3). This discrepancy might be attributable to the corn grain (concentrate) and timothy hay combination, which led to alterations in rumen fermentation (e.g., a higher molar proportion of propionate and lower methane production) as a result of microbial selection (Table 3). Therefore, to enable the wider application of seaweed extracts as a novel candidate additive in the future, the potential of seaweed extracts must be evaluated at various forage-to-concentrate ratios under in vitro and in vivo studies. Seaweed extract supplementation resulted in lower CO_2_ production than the CON at 12 and 48 h of incubation, which could indicate a high conversion rate of CO_2_ to CH_4_ by methanogenic archaea. Nevertheless, our results indicate that bioactive compounds in seaweed extracts are responsible for reducing methanogenic archaea to utilize free CO_2_ during methanogenesis, which could reduce CH_4_ production without decreasing methanogenic archaea (Tables 1, 3). Moreover, bioactive compounds such as phloroglucinol (monomeric unit of phlorotannins) decreased CH_4_ production with the reduction of methanogens^39^. Overall, our findings suggest that CH_4_ and CO_2_ decrease at a greater rate than the total gas production and this should direct more energy into VFA production.

In the rumen, it is known that CH_4_ production results from a mutualistic association between ciliate protozoa and methanogenic archaea^40^ (Table 3). Although there was no reduction of methanogenic archaea, a decrease in CH_4_ production may have been caused either by the suppressed metabolism of a CH_4_-producing microbe (independent of species) or the changed composition of the methanogenic community, or both^41^. Additionally, Zhou et al.^42^ reported that decreases in methanogenic archaea populations may not necessarily lead to a reduction in CH_4_ production, at least within short-term in vitro incubation. The discrepancy between CH_4_ production and the dynamics of the methanogenic archaea population might be partly attributable to the insensitivity of some ruminal methanogenic archaea to seaweed extracts.

A suitable compound for the reducing of methanogenesis in ruminants should be effective in reducing CH_4_ production and increasing propionate. The present study indicated that seaweed extracts can significantly decrease CH_4_ production, NH_3_-N concentrations, and shift the abundance of rumen microbial populations, but DM digestibility and total VFA production are not affected. Additionally, seaweed extracts possess the potential for CH_4_ reduction but do not always result in antiprotozoal activity suggesting that the unidirectional relationship between methanogenesis and protozoal numbers, as affected by seaweed extract, is not compulsory. Metagenomic and metabolomic approaches are essential for understanding how certain seaweed extracts impact the rumen microbiome and whether these effects hold promise as rumen modulators to improve rumen fermentation characteristics and productivity.

## Materials and methods

### Ethics statement

This study was performed in accordance with the principles of the Basel Declaration and recommendations of Laboratory Animals Guidelines of Gyeongsang National University (Jinju, Gyeongsangnam-do, Korea). All management and experimental protocols involving animals were approved by Gyeongsang National University Animal Research Ethical Committee (GNU-191011-E0050). This study followed standard procedures and ARRIVE guidelines to ensure an appropriate animal care.

### Brown seaweed extract preparation

The collection of seaweed material complied with institutional, national, and international guidelines and legislation concerning *Undaria pinnatifida* (*UPIN*), *Sargassum fusiforme* (*SFUS*), and *Sargassum fulvellum* (*SFUL*) seaweed. In accordance with guidelines and regulations for biosafety in Korea, extraction the seaweed (*UPIN*, *SFUS*, and *SFUL*), and residues was discarded according to protocol.

Three different types of brown seaweed extracts, i.e., *UPIN*, *SFUS*, and *SFUL* were purchased from the Jeju Biodiversity Research Institute (Jeju-do, Korea). Voucher specimens of the seaweed and other information regarding the seaweed extracts are available at this institute. Each fresh seaweed was cut or crushed into small pieces, freeze-dried and ground into a fine powder. Subsequently, powder was extracted with 80% (v/v) ethanol solvent (Daejung Chemical and Metals CO., Ltd, Siheung, Korea), and then placed in ultrasonic cleaner (Branson Ultrasonics Corporation, Danbury, CT, USA). Afterward, dimethylsulfoxide (Sigma-Aldrich Chemical Co., St. Louis, MO, USA) was infused to dissolve the stock solution (50-mg∙mL^−1^) of each extract and diluted using culture media.

### Chemical analysis

Chemical composition of substrates used on in vitro is shown in Table 4. Timothy hay and corn grain samples were ground through a 1 mm screen (Wiley Mill, Arthur Thomas Co., Philadelphia, PA), prior to in vitro and chemical analysis. Official methods of AOAC^43^ were used to analyze dry matter (DM), crude protein (CP, % of DM), ether extract (EE, % of DM) and Ash (% of DM) contents in the substrate. Neutral and acid detergent fiber (NDF and ADF, % of DM) contents were analyzed according to previously described method of Van Soest et al.^44^. Non-fibrous carbohydrate (NFC, % of DM) was calculated by following equation:

$${\text{NFC}} = {1}000 - \left( {{\text{CP}} + {\text{EE}} + {\text{Ash}} + {\text{NDF}}} \right).$$

**Table 4:** Chemical composition of substrates used in the in vitro experiment.

Item (% of DM)	Timothy hay	Corn grain
DM	93.82 ± 0.57	86.99 ± 0.59
CP	10.46 ± 0.39	9.32 ± 0.33
EE	5.48 ± 0.07	2.80 ± 0.54
Ash	5.93 ± 0.03	1.35 ± 0.01
NDF	62.27 ± 0.13	35.14 ± 1.03
ADF	38.14 ± 0.43	5.04 ± 0.05
Ca	0.21 ± 0.00	0.02 ± 0.00
P	0.11 ± 0.00	0.20 ± 0.00
NFC	15.86	51.40

All values represent the mean ± SEM (n = 3).

SEM Standard error of the mean, DM Dry matter, CP Crude protein, EE Ether extract, NDF Neutral detergent fiber, ADF Acid detergent fiber, NFC Non-fibrous carbohydrate.

Total flavonoid content was determined using the method of Zhishen et al.^45^ and Woisky and Salatino^46^ with slight modifications. In short, an aliquot of 100 μL of each seaweed extract solutions or standard (( +)-catechin hydrate) were mixed with 7.5 μL of sodium nitrite (5%), 15 μL of aluminium chloride (10%), 100 μL of 1 M sodium hydroxide, and 25 μL of distilled water and allowed to react 30 min. The total flavonoid concentration of each seaweed extracts was measured by microplate reader (SpectraMax M5, Molecular Devices, Sunnyvale, CA, USA) at 510 nm.

Total polyphenol content was determined using the method of Singleton et al.^47^ with minor modifications. Briefly, 100 μL of seaweed extracts or standard (gallic acid) were infused into an Eppendorf tube followed by 100 uL of 1 N Folin–Ciocalteu reagent solution. Afterward, 100 uL of 2% sodium carbonate solution was infused and tubes were thoroughly mixed by vortexing and allowed to stand for 30 min at room temperature. The total polyphenol concentration of each seaweed extracts was measured by microplate reader (SpectraMax M5, Molecular Devices, Sunnyvale, CA, USA) at 750 nm.

### Experiment procedures

Rumen fluid was collected from two non-lactating cannulated Hanwoo cows (average body weight = 440 kg) before morning feeding. The cows were fed a twice daily (0900 and 1700) for 2% DM of their body weight of timothy hay and commercial concentrate in a 6:4 ratios with free access to clean drinking water and a mineral block. Rumen fluid samples were filtered through four layers of cheesecloth, immediately transferred to the laboratory kept in a water bath at 39 °C. Afterward, filtered rumen fluid mixed with a buffer medium^48^ at a ratio of 1:2 (v/v) and maintained at anaerobic environment. Then, mixture (40 mL/bottle) was accurately infused into a 120 ml serum bottle, under a stream of O_2_-free N_2,_ containing 500 mg of substrate which was composed of 300 mg of timothy hay and 200 mg of corn grain; substrates were placed into nylon bags, which were later sealed and poured into the serum bottles. Seaweed extract mixtures were used at 1 dose amount: (0.25 mg∙mL^−1^) 5% of substrate of in vitro incubation medium. A CON (without seaweed extracts) was included in parallel. The dose amount (0.25 mg∙mL^−1^) was determined by previous studies^20,49^. Bottles were capped with a butyl rubber and placed in shaking incubator (120 rpm) at 39 °C for 6, 12, 24, 36 and 48 h incubation. A total of 115 bottles were used for 4 treatment including CON with 5 replicates each time points (6, 12, 24, 36 and 48 h incubation). The following treatments were used: (1) CON, (2) supplementation 0.25 mg∙mL^−1^ of *UPIN* extract, (3) supplementation 0.25 mg∙mL^−1^ of *SFUS* extract 5%, (4) supplementation 0.25 mg∙mL^−1^ of *SFUL* extract. There were also 15 bottles that were used for without substrate as a blank with 3 replicates each time points (6, 12, 24, 36 and 48 h incubation).

### Sampling and measurements

In vitro gas production during 6, 12, 24, 36 and 48 h incubation was determined by using a pressure transducer (Laurel Electronics, Inc., Costa Mesa, CA, USA) as described by Theodorou et al.^50^. All pressure values were converted to gas volume (mL) from the following equation defined by our laboratory conditions

$${\text{V}} = \left( {{\text{P}} - {11}.{271}} \right)/{8}.{5822}\left( {{\text{n}} = {144},{\text{R}}_{{2}} = 0.{999}} \right)$$

where V is gas volume (mL), P is measured pressure (psi).

Headspace gas (6 mL) was collected from each bottle and moved into vacuum test tube (Vacutainer, Becton Dickinson, Franklin Laker, NJ, USA). Concentration of CH_4_ and CO_2_ in the gas samples were determined by a gas chromatography (Shimadzu, GC-2010 PLUS, Japan) equipped with HP-PLOT Q capillary column (I.D. 0.53 mm, L.30 m) and flame ionization detector (FID). The temperature of the column, injector and detector were set at 50, 150 and 200 °C, respectively. Helium and H_2_ gases were used as carrier and combustion gases, respectively. The total production of CH_4_ and CO_2_ was calculated according to López et al.^51^ as follows:

$$\begin{aligned} & {\text{CH}}_{{4}} ,\;{\text{mL}} = {\text{CH}}_{{4}} {\text{concentration}}\left( {{\text{mL}}/{\text{mL}}} \right) \times \left( {{\text{Total}}\;{\text{gas}},{\text{mL}} + {\text{Headspace}},{8}0\;{\text{mL}}} \right) \\ & {\text{CO}}_{{2}} ,\;{\text{mL}} = {\text{ CO}}_{{2}} {\text{concentration}}\left( {{\text{mL}}/{\text{mL}}} \right) \times \left( {{\text{Total}}\;{\text{gas}},{\text{mL}} + {\text{Headspace}},{8}0\;{\text{mL}}} \right). \\ \end{aligned}$$

The pH value of each sample recorded using a pH meter (S220, Mettler-Toledo, Greifensee, Switzerland). A 2 mL of liquid sub-sample was collected from 6, 12, 24, 36 and 48 h incubations and then immediately centrifuged at 20,000 × g for 10 min at 4 °C for analyze NH_3_-N. The NH_3_-N was analyzed as described in Chaney and Marbach^52^, where the NH_3_-N concentration was adapted for 96 well plates with absorbance at 630 nm with a spectrometer (Model 680, Bio-Rad Laboratories, Hercules, CA, USA). A rumen fluid (2 mL) was collected from 12 and 24 h incubation and then immediately centrifuged at 20,000 × g for 10 min at 4 °C for analyze VFA. The VFA concentration was measured with a high performance liquid chromatography (L-2200, Hitachi, Tokyo, Japan) according to the method of Adesogan et al.^53^. Remaining rumen fluid (2 mL) of 24 h incubation was centrifuged at 20,000 × g for 15 min at 4 °C, supernatant was discarded, and the pellet was stored at − 80 °C in a freezer until use for DNA extraction and microbial community analysis. The apparent DM digestibility of the substrate was estimated after drying the residues collected in the nylon bags and the initial substrate at oven dried at 105 °C for 24 h.

### Microbial DNA extraction and quantitative real-time-polymerase chain reaction

Total DNA was extracted from the pellet stored at − 80 °C using the repeated bead beating plus column method^54^. Genomic DNA was extracted from triplicate samples using QIAamp Fast DNA Stool Mini Kit (Qiagen, Hilden, Germany) following manufacturer recommendations. The quality and quantity of extracted DNA were analyzed with a NanoDrop ND-2000 spectrophotometer (Thermo Fisher Scientific Inc., Waltham, MA, USA). Real-time quantitative PCR assays for enumeration of total bacteria, ciliate protozoa, fungi, methanogenic archaea, fibrolytic bacteria (*Fibrobacter succinogenes*, *Ruminococcus albus*, *Ruminococcus flavefaciens*), proteolytic bacteria (*Butyrivibrio fibrisolvens*, *Butyrivibrio proteoclasticus*, *Prevotella ruminicola*, *Anaerovibrio lipolytica*) were conducted as described by Denman and McSweeney^55^ and Khafipour^56^ on a CFX 96 Touch system (Bio-Rad Laboratories). Specific information on primer sequences for rumen microbes is presented in Table 5. All the reaction were carried out triplicate, total reaction volumes of 20 μL. Reaction mixture consisted of 0.5 μL of 10 mM dNTP mix (BioFACT, Daejeon, Korea), 2 μL of 10 × buffer (BioFACT, Daejeon, Korea), 1 μL of tenfold diluted genomic DNA, each 1 1 μL of 10 μM primer-set, 0.1 μL of taq polymerase (BioFACT, Daejeon, Korea), 1 μL of Evagreen (SolGent, Daejeon, Korea), and 13.4 μL of bio-grade water. For absolute quantification of each microbes, using a standard plasmid DNA to the respective target sequence. All the detail procedure of PCR condition and manufacturing each microbe plasmids were proceeded according to Kim et al.^57^ and Hamid et al.^58^.

**Table 5:** Primers (F = forward, R = reverse) for real-time PCR assay.

Target species	Primer sequence (5′ → 3′)	Size (bp)^a^	Efficiency^b^	References
Total bacteria	F: CGGCAACGAGCGCAACCC R: CCATTGTAGCACGTGTGTAGCC	130	1.98	^56,55^
Ciliate protozoa	F: GCTTTCGWTGGTAGTGTATT R: CTTGCCCTCYAATCGTWCT	223	1.90	^59^
Fungi	F: GAGGAAGTAAAAGTCGTAACAAGGTTTC R: CAAATTCACAAAGGGTAGGATGATT	120	1.84	^55^
Methanogenic archaea	F: GAGGAAGGAGTGGACGACGGTA R: ACGGGCGGTGTGTGCAAG	232	2.08	^59^
*Fibrobacter succinogenes*	F: GTTCGGAATTACTGGGCGTAAA R: CGCCTGCCCCTGAACTATC	121	1.88	^55^
*Ruminococcus albus*	F: CCCTAAAAGCAGTCTTAGTTCG R: CCTCCTTGCGGTTAGAACA	176	1.84	^60^
*Ruminococcus flavefaciens*	F: CGAACGGAGATAATTTGAGTTTACTTAGG R: CGGTCTCTGTATGTTATGAGGTATTACC	132	1.81	^55^
*Butyrivibrio fibrisolvens*	F: ACCGCATAAGCGCACGGA R: CGGGTCCATCTTGTACCGATAAAT	78	1.89	^61^
*Butyrivibrio proteoclasticus*	F: TCCGGTGGTATGAGATGGGC R: GTCGCTGCATCAGAGTTTCCT	65	2.17	^62^
*Prevotella ruminicola*	F: GCGAAAGTCGGATTAATGCTCTATG R: CCCATCCTATAGCGGTAAACCTTTG	185	2.04	^56^
*Anearovibrio lipolytica*	F: TGGGTGTTAGAAATGGATTC R: CTCTCCTGCACTCAAGAATT	597	1.83	^63^

^a^bp, base pair, ^b^Efficiency is calculated as [10^−1/slope^].

### NMR spectroscopy and metabolite identification and quantification

Five hundred microliters of methanol-*d4*, 400 μL of 0.2 M phosphate buffer solution (0.2 M of sodium hydrogen phosphate, 0.2 M sodium dihydrogen phosphate in D_2_O, pH 7.0 ± 0.1), and 100 μL of 5 mM TSP (3-trimethylsilyl propionic-2, 2, 3, 3-*d4* acid sodium salt) were added into an Eppendorf tube containing 50 ± 0.5 mg of each seaweed extract. ^1^H-NMR experiments were carried out on Ascend 800 MHz, Avance III HD Bruker spectrometer (Bruker Biospin AG, Fällanden, Switzerland) equipped with 5 mm CPTIC ^1^H-^13^C/15 N/D Z-GRD Z119427/0011 cryogenic probe. ^1^H-NMR spectra were processed and analysed using Chenomx NMR Suite 8.4 (Chenomx, Edmonton, AB, Canada). All ^1^H-NMR spectra were calibrated, phased and baseline-corrected manually using the processor module of Chenomx NMR Suite. The concentration and profiling of metabolites were estimated using the profiler module of Chenomx NMR Suite. The detail procedures of such analyses has been reported in a previous article Choi et al.^23^.

### Statistical analysis

The online open-source platform, MetaboAnalyst 5.0 was used for the multivariate analyses including principal component analysis (PCA) and partial least squares-discriminant analysis (PLS-DA) of metabolites in seaweed extracts. To refine analysis of metabolites in seaweed extracts, the variable importance in projection (VIP) score along the predictive component were acquired. The VIP score exceeding 1.5 were selected as differentially expressed metabolites. Detail procedures of such analyses has been reported in a previous article^64^. The data of in vitro was statistically analyzed using the PROC MIXED procedure of SAS 9.4. The statistical model used in this study as follow:

$${\text{Y}}_{ijk} = \mu + t_{i} + r_{j} + \gamma_{ijk}$$

where Y_*ijk*_ is the experimental data; *µ* is the overall mean, *t*_*i*_ is the fixed effect of dietary treatments; *r*_*j*_ is the random effect of replication; and *γ*_*k*_ is the unexplained random error. Tukey’s multiple range test was used to identify differences between treatments. Statistical significance was declared at *P* ≤ 0.05, and a trend was discussed when 0.05 < *P* ≤ 0.10.

## References

[1] Stocker, T. F. *et al.* Climate change 2013. The physical science basis. Working group I contribution to the fifth assessment report of the intergovernmental panel on climate change-abstract for decision-makers; Changements climatiques 2013. Les elements scientifiques. Contribut. (2013).

[2] Zhao L (2020). Ozone decreased enteric methane production by 20% in an in vitro rumen fermentation system. Front. Microbiol..

[3] Johnson KA, Johnson DE (1995). Methane emissions from cattle. J. Anim. Sci..

[4] Odongo NE (2007). Long-term effects of feeding monensin on methane production in lactating dairy cows. J. Dairy Sci..

[5] Immig I, Demeyer D, Fiedler D, Van Nevel C, Mbanzamihigo L (1996). Attempts to induce reductive acetogenesis into a sheep rumen. Arch. Anim. Nutr..

[6] Kim SH, Mamuad LL, Kim DW, Kim SK, Lee SS (2016). Fumarate reductase-producing enterococci reduce methane production in rumen fermentation in vitro. J. Microbiol. Biotechnol..

[7] Patra AK, Yu Z (2012). Effects of essential oils on methane production and fermentation by, and abundance and diversity of, rumen microbial populations. Appl. Environ. Microbiol..

[8] Kinley RD (2020). Mitigating the carbon footprint and improving productivity of ruminant livestock agriculture using a red seaweed. J. Clean. Prod..

[9] Roque BM (2019). Effect of the macroalgae *Asparagopsis taxiformis* on methane production and rumen microbiome assemblage. Anim. Microbiome.

[10] Wang Y, Xu Z, Bach SJ, McAllister TA (2008). Effects of phlorotannins from *Ascophyllum nodosum* (brown seaweed) on in vitro ruminal digestion of mixed forage or barley grain. Anim. Feed Sci. Technol..

[11] Wang Y, Alexander TW, Mcallister TA (2009). In vitro effects of phlorotannins from *Ascophyllum nodosum* (brown seaweed) on rumen bacterial populations and fermentation. J. Sci. Food Agric..

[12] Wood JM, Kennedy FS, Wolfe RS (1968). Reaction of multihalogenated hydrocarbons with free and bound reduced vitamin B12. Biochemistry.

[13] Allen KD, Wegener G, White RH (2014). Discovery of multiple modified F430 coenzymes in methanogens and anaerobic methanotrophic archaea suggests possible new roles for F430 in nature. Appl. Environ. Microbiol..

[14] Li Y-X, Wijesekara I, Li Y, Kim S-K (2011). Phlorotannins as bioactive agents from brown algae. Process Biochem..

[15] Belanche A, Jones E, Parveen I, Newbold CJ (2016). A metagenomics approach to evaluate the impact of dietary supplementation with *Ascophyllum nodosum* or Laminaria digitata on rumen function in Rusitec fermenters. Front. Microbiol..

[16] Scalbert A (1991). Review article number 63 antimicrobial properties of tannins. Phytochemistry.

[17] Choi YY (2020). In vitro and in situ evaluation of *Undaria pinnatifida* as a feed ingredient for ruminants. J. Appl. Phycol..

[18] Choi YY (2020). The potential nutritive value of *Sargassum fulvellum* as a feed ingredient for ruminants. Algal Res..

[19] Choi YY (2020). New challenges for efficient usage of *Sargassum fusiforme* for ruminant production. Sci. Rep..

[20] Choi YY (2021). In vitro five brown algae extracts for efficiency of ruminal fermentation and methane yield. J. Appl. Phycol..

[21] Machado L, Magnusson M, Paul NA, De Nys R, Tomkins N (2014). Effects of marine and freshwater macroalgae on in vitro total gas and methane production. PLoS ONE.

[22] Belanche A, Ramos-Morales E, Newbold CJ (2016). In vitro screening of natural feed additives from crustaceans, diatoms, seaweeds and plant extracts to manipulate rumen fermentation. J. Sci. Food Agric..

[23] Maia MRG, Fonseca AJM, Oliveira HM, Mendonça C, Cabrita ARJ (2016). The potential role of seaweeds in the natural manipulation of rumen fermentation and methane production. Sci. Rep..

[24] Makkar HPS (2003). Effects and fate of tannins in ruminant animals, adaptation to tannins, and strategies to overcome detrimental effects of feeding tannin-rich feeds. Small Rumin. Res..

[25] Stewart CS, Flint HJ, Bryant MP, Hobson PN, Stewart CS (1997). The rumen bacteria. The Rumen Microbial Ecosystem.

[26] Andries JI, Buysse FX, De Brabander DL, Cottyn BG (1987). Isoacids in ruminant nutrition: Their role in ruminal and intermediary metabolism and possible influences on performances—A review. Anim. Feed Sci. Technol..

[27] Wettstein H-R, Machmüller A, Kreuzer M (2000). Effects of raw and modified canola lecithins compared to canola oil, canola seed and soy lecithin on ruminal fermentation measured with rumen simulation technique. Anim. Feed Sci. Technol..

[28] Kobayashi Y (2010). Abatement of methane production from ruminants: Trends in the manipulation of rumen fermentation. Asian-Australas. J. Anim. Sci..

[29] Henderson C (1980). The influence of extracellular hydrogen on the metabolism of *Bacteroides ruminicola*, *Anaerovibrio lipolytica* and *Selenomonas ruminantium*. J. Gen. Microbiol..

[30] Becker PM (2014). Evidence for a hydrogen-sink mechanism of (+)catechin-mediated emission reduction of the ruminant greenhouse gas methane. Metabolomics.

[31] AlZahal O, Li F, Guan LL, Walker ND, McBride BW (2017). Factors influencing ruminal bacterial community diversity and composition and microbial fibrolytic enzyme abundance in lactating dairy cows with a focus on the role of active dry yeast. J. Dairy Sci..

[32] Bauman, D. E., Perfield, J. W., De Veth, M. J. & Lock, A. L. New perspectives on lipid digestion and metabolism in ruminants. In *Proceedings of Cornell Nutrition Conference*, vol. 65, 175–189 (Cornell University, 2003).

[33] Lee SY (2011). Glycerol as a feed supplement for ruminants: In vitro fermentation characteristics and methane production. Anim. Feed Sci. Technol..

[34] Janssen PH, Kirs M (2008). Structure of the archaeal community of the rumen. Appl. Environ. Microbiol..

[35] O’Hara E, Neves ALA, Song Y, Guan LL (2020). The role of the gut microbiome in cattle production and health: Driver or passenger?. Annu. Rev. Anim. Biosci..

[36] Henderson G (2015). Rumen microbial community composition varies with diet and host, but a core microbiome is found across a wide geographical range. Sci. Rep..

[37] Molina-Alcaide E (2017). In vitro ruminal fermentation and methane production of different seaweed species. Anim. Feed Sci. Technol..

[38] Zhou M (2018). Air-dried brown seaweed, *Ascophyllum nodosum*, alters the rumen microbiome in a manner that changes rumen fermentation profiles and lowers the prevalence of foodborne pathogens. mSphere.

[39] Sarwono KA, Kondo M, Ban-Tokuda T, Jayanegara A, Matsui H (2019). Effects of phloroglucinol and the forage: Concentrate ratio on methanogenesis, in vitro rumen fermentation, and microbial population density. Adv. Anim. Vet. Sci.

[40] Belanche A, de la Fuente G, Newbold CJ (2014). Study of methanogen communities associated with different rumen protozoal populations. FEMS Microbiol. Ecol..

[41] Machmüller A, Soliva CR, Kreuzer M (2003). Effect of coconut oil and defaunation treatment on methanogenesis in sheep. Reprod. Nutr. Dev..

[42] Zhou M (2011). Relationship between rumen methanogens and methane production in dairy cows fed diets supplemented with a feed enzyme additive. J. Appl. Microbiol..

[43] AOAC. Official methods of analysis. In *Association of Official Analytical Chemists*, 15th edn. (1990).

[44] Van Soest PJ, Robertson JB, Lewis BA (1991). Symposium: Carbohydrate methodology, metabolism, and nutritional implications in dairy cattle. J. Dairy Sci..

[45] Zhishen J, Mengcheng T, Jianming W (1999). The determination of flavonoid contents in mulberry and their scavenging effects on superoxide radicals. Food Chem..

[46] Woisky RG, Salatino A (1998). Analysis of propolis: Some parameters and procedures for chemical quality control. J. Apic. Res..

[47] Singleton VL, Orthofer R, Lamuela-Raventós RM (1999). [14] Analysis of total phenols and other oxidation substrates and antioxidants by means of folin-ciocalteu reagent. Methods Enzymol..

[48] McDougall EI (1948). Studies on ruminant saliva. 1. The composition and output of sheep’s saliva. Biochem. J..

[49] Lee SJ (2018). Effect of Rhodophyta extracts on in vitro ruminal fermentation characteristics, methanogenesis and microbial populations. Asian-Australasian J. Anim. Sci..

[50] Theodorou MK, Williams BA, Dhanoa MS, McAllan AB, France J (1994). A simple gas production method using a pressure transducer to determine the fermentation kinetics of ruminant feeds. Anim. Feed Sci. Technol..

[51] López S (2007). Some methodological and analytical considerations regarding application of the gas production technique. Anim. Feed Sci. Technol..

[52] Chaney AL, Marbach EP (1962). Modified reagents for determination of urea and ammonia. Clin. Chem..

[53] Adesogan AT, Krueger N, Salawu MB, Dean DB, Staples CR (2004). The influence of treatment with dual purpose bacterial inoculants or soluble carbohydrates on the fermentation and aerobic stability of bermudagrass. J. Dairy Sci..

[54] Yu Z, Morrison M (2004). Improved extraction of PCR-quality community DNA from digesta and fecal samples. Biotechniques.

[55] Denman SE, McSweeney CS (2006). Development of a real-time PCR assay for monitoring anaerobic fungal and cellulolytic bacterial populations within the rumen. FEMS Microbiol. Ecol..

[56] Khafipour E, Li S, Plaizier JC, Krause DO (2009). Rumen microbiome composition determined using two nutritional models of subacute ruminal acidosis. Appl. Environ. Microbiol..

[57] Kim H, Kim B, Cho S, Kwon I, Seo J (2020). Dietary lysophospholipids supplementation inhibited the activity of lipolytic bacteria in forage with high oil diet: An in vitro study. Asian-Australas. J. Anim. Sci..

[58] Hamid MMA (2021). Rumen fermentation, methane production, and microbial composition following in vitro evaluation of red ginseng byproduct as a protein source. J. Anim. Sci. Technol..

[59] Sylvester JT, Karnati SKR, Yu Z, Morrison M, Firkins JL (2004). Development of an Assay to Quantify Rumen Ciliate Protozoal Biomass in Cows Using Real-Time PCR. J. Nutr..

[60] Wang RF, Cao WW, Cerniglia CE (1997). A universal protocol for PCR detection of 13 species of foodborne pathogens in foods. J. Appl. Microbiol..

[61] Stevenson DM, Weimer PJ (2007). Dominance of Prevotella and low abundance of classical ruminal bacterial species in the bovine rumen revealed by relative quantification real-time PCR. Appl. Microbiol. Biotechnol..

[62] Paillard D (2007). Relation between phylogenetic position, lipid metabolism and butyrate production by different Butyrivibrio-like bacteria from the rumen. Antonie van Leeuwenhoek. Int. J. Gen. Mol. Microbiol..

[63] Tajima K (2001). Diet-Dependent Shifts in the Bacterial Population of the Rumen Revealed with Real-Time PCR. Appl. Environ. Microbiol..

[64] Eom JS (2021). Metabolomics comparison of rumen fluid and milk in dairy cattle using proton nuclear magnetic resonance spectroscopy. Anim. Biosci..

